# Fetal hemodynamics and cardiac streaming assessed by 4D flow cardiovascular magnetic resonance in fetal sheep

**DOI:** 10.1186/s12968-018-0512-5

**Published:** 2019-01-21

**Authors:** Eric M. Schrauben, Brahmdeep Singh Saini, Jack R. T. Darby, Jia Yin Soo, Mitchell C. Lock, Elaine Stirrat, Greg Stortz, John G. Sled, Janna L. Morrison, Mike Seed, Christopher K. Macgowan

**Affiliations:** 1 Translational Medicine, Hospital for Sick Children, Toronto, Canada; 2 Heart Centre, Hospital for Sick Children, Toronto, Canada; 3 Institute of Medical Science, Faculty of Medicine, University of Toronto, Toronto, Canada; 4 Early Origins of Adult Health Research Group, School of Pharmacy and Medical Sciences, Sansom Institute for Health Research, University of South Australia, Adelaide, Australia; 5 Department of Medical Biophysics, University of Toronto, Toronto, Canada; 6 Division of Cardiology, Hospital for Sick Children, Toronto, Canada; 7 Department of Paediatrics, University of Toronto, Toronto, Canada

**Keywords:** Fetal, Cardiac, Hemodynamics, 4D flow CMR, Cardiovascular magnetic resonance

## Abstract

**Background:**

To date it has not been possible to obtain a comprehensive 3D assessment of fetal hemodynamics because of the technical challenges inherent in imaging small cardiac structures, movement of the fetus during data acquisition, and the difficulty of fusing data from multiple cardiac cycles when a cardiac gating signal is absent. Here we propose the combination of volumetric velocity-sensitive cardiovascular magnetic resonance imaging (“4D flow” CMR) and a specialized animal preparation (catheters to monitor fetal heart rate, anesthesia to immobilize mother and fetus) to examine fetal sheep cardiac hemodynamics in utero.

**Methods:**

Ten pregnant Merino sheep underwent surgery to implant arterial catheters in the target fetuses. Anesthetized ewes underwent 4D flow CMR with acquisition at 3 T for fetal whole-heart coverage with 1.2–1.5 mm spatial resolution and 45–62 ms temporal resolution. Flow was measured in the heart and major vessels, and particle traces were used to visualize circulatory patterns in fetal cardiovascular shunts. Conservation of mass was used to test internal 4D flow consistency, and comparison to standard 2D phase contrast (PC) CMR was performed for validation.

**Results:**

Streaming of blood from the ductus venosus through the foramen ovale was visualized. Flow waveforms in the major thoracic vessels and shunts displayed normal arterial and venous patterns. Combined ventricular output (CVO) was 546 mL/min per kg, and the distribution of flows (%CVO) were comparable to values obtained using other methods. Internal 4D flow consistency across 23 measurement locations was established with differences of 14.2 ± 12.1%. Compared with 2D PC CMR, 4D flow showed a strong correlation (R^2^ = 0.85) but underestimated flow (bias = − 21.88 mL/min per kg, *p* < 0.05).

**Conclusions:**

The combination of fetal surgical preparation and 4D flow CMR enables characterization and quantification of complex flow patterns in utero. Visualized streaming of blood through normal physiological shunts confirms the complex mechanism of substrate delivery to the fetal heart and brain. Besides offering insight into normal physiology, this technology has the potential to qualitatively characterize complex flow patterns in congenital heart disease phenotypes in a large animal model, which can support the development of new interventions to improve outcomes in this population.

**Electronic supplementary material:**

The online version of this article (10.1186/s12968-018-0512-5) contains supplementary material, which is available to authorized users.

## Background

**Fig. 1:**
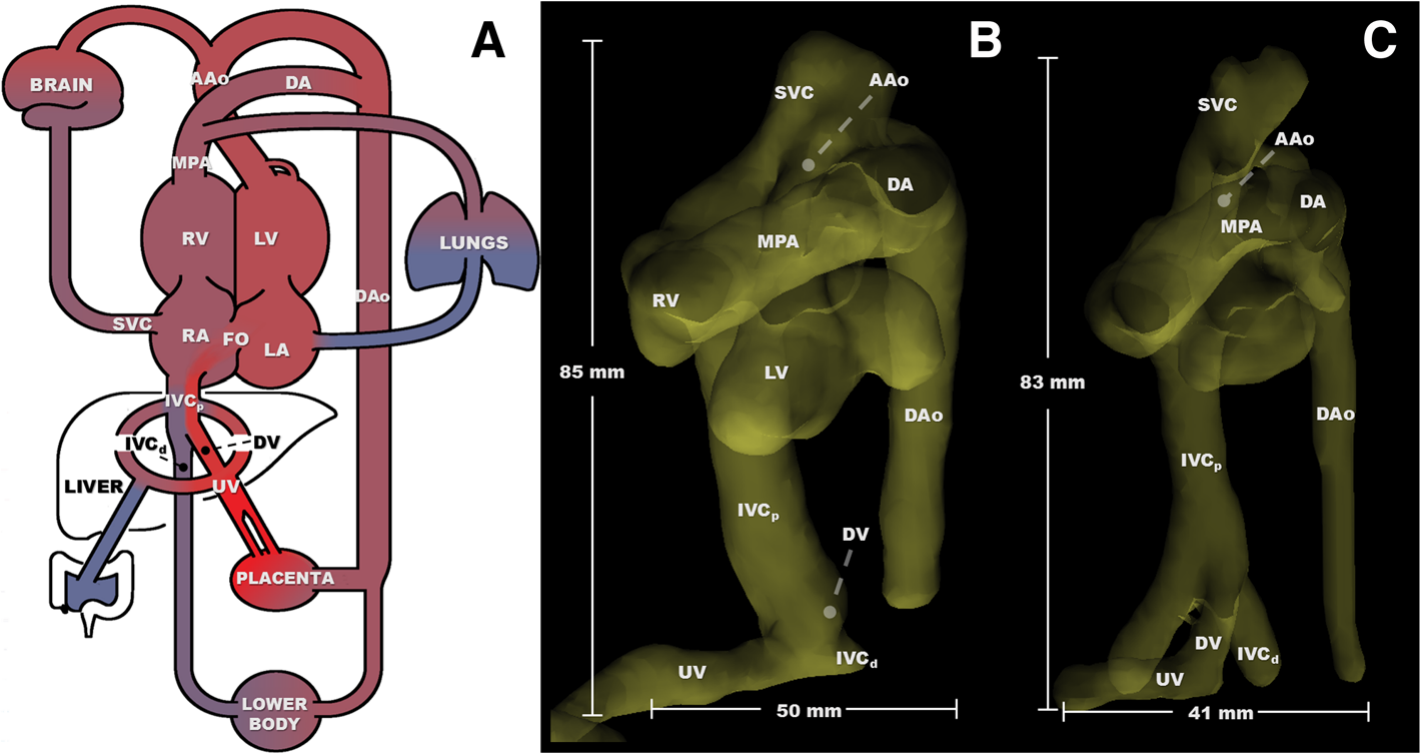
**a**: Normal course of the fetal circulation. **b**, **c**: Two examples of segmented whole-heart angiographic images in the left ventral view, derived from time-averaged phase contrast data (gestational age: B = 128 days, C = 116 days). Scale bars indicate relative size of fetal structures, with standard anatomy labeled. UV: umbilical vein; DV: ductus venosus; IVC_d_: distal inferior vena cava; IVC_p_: proximal inferior vena cava; SVC: superior vena cava; RA: right atrium; FO: foramen ovale; LA: left atrium; RV: right ventricle; LV: left ventricle; MPA: main pulmonary artery; DA: ductus arteriosus; AAo: ascending aorta; DAo: descending aorta

The fetal circulatory system differs from the post-natal circulation of placental mammals due to the need to exchange oxygen, nutrients, and waste across the placenta – the presence of shunts in the fetal circulation creates additional paths by which oxygen-rich blood transits directly to the heart and brain. Early investigations explored the fetal circulation by injecting radiolabeled microspheres into target vessels and measuring their deposition in downstream tissues [1, 2]. These studies revealed remarkable patterns of flow within the fetus, including the preferential delivery of oxygenated umbilical vein blood to critical organs. This is made possible by two physiological shunts unique to the fetal circulation: the ductus venosus and foramen ovale (Fig. 1a). The ductus venosus provides a route for some oxygen- and nutrient-rich blood from the umbilical vein to bypass the liver and flow directly into the proximal inferior vena cava (IVC_p_). This blood then flows preferentially through the right atrium and into the left atrium, via the foramen ovale, to eventually supply the myocardium, brain and upper body. Conversely, these same studies showed that deoxygenated and nutrient poor blood from the distal inferior vena cava (IVC_d_) and superior vena cava (SVC) flows preferentially from the right atrium into the right ventricle, which primarily supplies the placenta and lower body via the ductus arteriosus. This confluence and subsequent separation of oxygenated and deoxygenated blood, with limited mixing in the IVC_p_ and right atrium, is now recognized as essential for normal fetal development, and its disruption in congenital heart disease affects growth and development [3–5].

Despite the importance of this complex flow network, it is difficult to visualize and quantify in vivo. Recent imaging studies of the fetal circulation have used ultrasound to measure velocity waveforms [6, 7] and single-slice two-dimensional (2D) phase-contrast (PC) CMR to quantify blood flow in target vessels in humans [8, 9] and sheep [10]. While these studies have elucidated important aspects of fetal circulation, the complex course of oxygenated and deoxygenated blood flow to and through the fetal heart has not been directly observed and mapped. Understanding normal cardiovascular physiology may improve detection of abnormal development to design new intervention strategies appropriate for clinical intervention. 4D flow CMR [11], an extension of 2D PC CMR that quantifies cardiac time-resolved three dimensional (3D) blood flow within a prescribed anatomical volume, is ideally suited to capture these fetal cardiovascular dynamics. Resulting velocity fields can be visualized and interactively probed using particle traces (paths followed by virtual particles released into the time-dependent velocity field).

The purpose of this work was to examine 3D fetal cardiac hemodynamics in utero. This is accomplished using the combination of specialized animal preparation – surgical placement of pressure catheters to measure fetal heart rate and anesthesia to immobilize the mother and fetus – and 4D flow CMR of the fetal heart. We present our initial experience in a sheep model of human pregnancy, as well as qualitative and quantitative findings with reference to 2D PC CMR findings.

## Materials and methods

All procedures were approved by the South Australian Health and Medical Research Institute’s Animal Ethics Committee and complied with the Australian code of practice for the care and use of animals for scientific purposes. All investigators understood and followed the ethical principles outlined in Grundy et al. [12] as well as the ARRIVE guidelines [13].

### Animal care and preparation

This study included 10 fetuses from 10 pregnant Merino ewes (singleton pregnancies, full term = 150 days). Ewes were housed in individual pens in animal holding rooms with a 12:12 h light/dark cycle and were fed 100% metabolizable energy requirements once daily with ad libitum access to water. The day before surgery, ewes were administered meloxicam (subcutaneously; 0.5 mg/kg) and again 24 h later. Each ewe and fetus underwent surgery at 109–111 days gestation to implant vascular catheters. General anesthesia was induced with intravenous diazepam (0.3 mg/kg) and ketamine (5 mg/kg) and maintained with 1–2% isoflurane in 100% oxygen (Lyppards, South Australia, Australia). An incision was made in the ewes’ abdomen and the uterus was opened to expose the fetus. Vascular catheters (Critchley Electrical Products, Silverwater, Australia) were inserted into a fetal femoral artery, jugular vein and the amniotic cavity. The fetus was returned to the uterus, and the uterine and abdominal incisions were sutured closed [5, 10, 14, 15]. At the time of surgery, ewes and fetuses received an intramuscular injection of antibiotics of 3.5 mL of Norocillin – 150 mg/ml procaine penicillin and 112.5 mg/mL benzathine penicillin (Norbrook Laboratories Ltd., Gisborne, Australia) and 2 mL of 125 mg/mL Dihydrostreptomycin in sterile saline (Sigma, St Louis, Missouri, USA). These were administered to ewes intramuscularly for 3 days post-op and fetuses received ampicillin intraamniotically for 4 days. Animals were allowed at least 4 days to recover from surgery prior to experiments.

### Monitoring fetal health

Fetal arterial blood was collected daily to monitor fetal health. Measurement of whole blood arterial partial pressure of oxygen, partial pressure of carbon dioxide, pH, oxygen saturation, hematocrit, and hemoglobin were measured with a RAPIDPOINT 500 (Siemens Healthineers, Erlangen, Germany) and temperature corrected to 39 °C.

### CMR animal preparation

General anesthesia was induced at 123 ± 7 days gestation with intravenous diazepam (0.3 mg/kg) and ketamine (5 mg/kg) and maintained with 2.5–3.0% isoflurane. The ewe was then positioned on its left side for the duration of the scan, with respiration supported by ventilator with room air and condition monitored with an CMR compatible SaO_2_/heart rate monitor (Nonin Medical Inc., Plymouth, Minnesota, USA).

The fetal femoral artery and amniotic catheters were connected to displacement transducers, a quad-bridge amplifier and a data acquisition unit (PowerLab, ADInstruments, Castle Hill, Australia) to record fetal blood pressure (corrected for amniotic pressure). All data were sampled at a rate of 400 Hz, digitized and recorded using LabChart 7 (AD Instruments). Cardiac triggers were determined when the resulting blood pressure signal passed a threshold value, calibrated before scanning began. Individual triggers were sent in realtime during fetal scanning via the CMR external triggering port [5, 16].

### Imaging protocol

Following high resolution 3D imaging for cardiac localization, 4D flow CMR (WIP 785A, Siemens) was performed using a commercial 3 T CMR system (Magnetom Skyra, Siemens Healthineers). Data were acquired in an axial orientation relative to the fetus using 60 slices with coverage from the intra-hepatic umbilical vein to the aortic arch. To provide better velocity to noise contrast in slow flow regions, 6 subjects had 4D flow CMR repeated using the same volumetric prescription with high and low velocity encodings (two VENC; Table 1). All images were retrospectively cardiac gated and reconstructed to 8 cardiac timeframes using parallel imaging (acceleration factor = 2, 32 reference lines). The acquisition did not employ respiratory navigator gating or multiple averages at the same VENC..

**Table 1:** CMR acquisition and reconstruction parameters

CMR Acquisition and Reconstruction parameters		
	4D flow [Range]	2D PC [Range]
FOV (cm)	26–35 × 26–40 × 7.2–9.0	24 × 16.6
Spatial resolution (mm)	1.2–1.5 × 1.2–1.5 × 1.3–1.5	1.0 × 1.0 × 5.0
TR (ms)	8.2–10.9	5.59
TE (ms)	3.1–4.1	3.18
α (°)	8	30
Cardiac Frames	8	15
Reconstructed temporal resolution (ms)	45–62	24–33
Acquisition time (min)	6.5–8 (one VENC, 3 subjects);	1.83 (3 averages)
	15–19 (two VENC, 6 subjects)	
VENC (cm/s)	100–150 (one VENC, 4 subjects);	50–150
	50,150 (two VENC, 6 subjects)	

*FOV* field-of-view; *TE* echo time; *TR* repetition time; *VENC* velocity encoding

High resolution 2D PC CMR of the major fetal vessels was also performed. Images were acquired perpendicular to the vessel of interest, which included the ascending aorta (AAo), main pulmonary artery (MPA),ductus arteriosus, descending aorta (DAo), SVC, ductus venosus, left / right pulmonary arteries (LPA, RPA) and umbilical vein. Acquisition parameters in these scans can be found in Table 1. In the event of aliasing or non-orthogonal vessel prescriptions, the vessel of interest was re-localized and the measurement was repeated.

### Image processing

4D flow CMR data were processed and analyzed by an experienced researcher. After background phase subtraction based on the phase of static tissue, automatic phase unwrapping using the highest available VENC scan was employed to reduce phase aliasing [17]. Phase images from the two VENC acquisitions were combined on a voxel by voxel basis, selecting the low VENC velocity vector if phase aliasing was not evident when compared with the unwrapped high VENC scan [18].

Corrected data were subsequently processed using prototype visualization and analysis software (4D Flow v2.4, Siemens Healthineers) [19]. Briefly, whole-heart anatomy was segmented from the time-averaged PC angiogram using the vessel centerline tree and the vessel lumen extracted semi-automatically from user-placed seed points. Temporal motion tracking of flow data using symmetric deformable registration was applied and later used to temporally update the segmentation, analysis planes, and visualization of particle traces.

### Flow visualization

By defining specific fetal vessels as the origins of particle traces, the evolution of blood flow was visualized – here examining the circulatory mechanisms responsible for fetal oxygen delivery. Particles were emitted from cross-sections of the MPA and the AAo to visualize flow through the ductus arteriosus, aortic isthmus, and supply to the cerebrovascular circulation. Similarly, flow into the right heart was visualized via particle traces emitted from the SVC and IVC_p_. Finally, particles were emitted from the ductus venosus and IVC_d_ to trace the paths of blood returning from the placenta and lower fetal body, respectively.

### Flow quantification

4D flow CMR blood flow distribution was measured in all visible major vessels and flow conduits by placing cardiac time-resolved contours. To ensure these contours were orthogonal to the vessel and direction of flow, 3D velocity vectors were visualized within an initial contour, which was adjusted until vectors were perpendicular to the cross-section. Values were averaged over the cardiac cycle, indexed to fetal body weight (mL/min per kg), and used to calculate combined ventricular output (CVO = 1.03*[MPA + AAo]) and %CVO for each vessel [20].

Conservation of mass, which requires equal total flow entering and exiting a flow network, was used to test internal consistency of 4D flow CMR. Mean percent difference across four cardiovascular locations was calculated in cases where all vessels could be identified. These are illustrated in Additional file 1: Fig. S1: IVC_p_ versus combined right hepatic vein, ductus venosus and IVC_d_ (IVC_p_ = RHV + DV (ductus venosus) + IVC_d_; *n* = 7); flows to and from the right side of the heart (SVC + IVC_p_ = MPA + FO (foramen ovale); *n* = 4); MPA versus combined ductus arteriosus and pulmonary blood flow (MPA = DA + PBF; n = 4); and total flow to and from the lower body (IVC_p_ = DAo; *n* = 8). Pulmonary blood flow (PBF) was calculated as left pulmonary artery plus right pulmonary artery (PBF = LPA + RPA).

4D flow CMR and 2D PC CMR results were compared using indexed mean flows. Over all subjects and vessels where both techniques were successfully completed, least squares regression, Bland-Altman analysis [21], and a paired t-test were performed. The significance level was set to 5% (*p* ≤ 0.05).

Finally, at each measurement location, flows (absolute and indexed) were compared to those in the literature obtained using a variety of techniques and species: 2D PC CMR in humans [9], Doppler ultrasound in mice [7], radiolabeled microspheres in sheep [20], and 2D PC CMR in sheep [10].

## Results

### Animal characteristics and blood gas data

Fetal weight (calculated from fetal volume determined at CMR) and heart rate were appropriate for gestational age (Table 2). Fetal blood gases during the CMR session were also normal.

**Table 2:** Fetal characteristics and blood gas status at the time of CMR scanning

Gestational Age (days)	123	±	7
Weight (kg)	2.8	±	0.6
Heart rate (bpm)	140	±	13
Fetal blood gases			
Partial pressure of oxygen (mmHg)	17.8	±	2.9
Partial pressure of carbon dioxide (mmHg)	52.3	±	13.9
pH	7.287	±	0.058
Oxygen saturation (%)	51.1	±	7.1
Hematocrit (%)	26.7	±	4.5
Hemoglobin (g/L)	83.4	±	24.0

*Fetal body weight at CMR was estimated based on fetal volume in CMR* [22]*. [Mean ± StdDev]*

### Visualization and shunt quantification

Anatomic 3D segmentation of all target vessels was achieved for 5 of the 10 subjects, including all vessels listed in Fig. 1a. Example segmentations in two fetal sheep at different gestational ages are shown in Fig. 1b-c, including labeled vessels and shunts as well as scale bars for relative size. In the remaining 5 cases, at least 2 fetal vessels were segmented for flow measurement. Table 3 lists the number of times each vessel was measured across this cohort.

**Table 3:** Literature-based flow comparisons

		Method	CVO	MPA	AAo	SVC	DA	PBF^a^	DAo	UV	FO^b^	IVC_d_	IVC_p_^c^	DV
Mean Flow (mL/min per kg)	Human	2D PC MRI	465	261	191	137	187	74	252	134	135	–	–	–
	Mouse	ultrasound	1020	550	470	210	340	210	410	130	270	–	290	90
	Sheep	microspheres	450	300	137	108	260	36	305	180	115	125	305	100
	Sheep	2D PC MRI	517	266	236	121	200	89	352	213	164	–	–	105
	Sheep	4D flow MRI	546	284	245	181	265	38	333	197	174	117	357	144
	# measurements			6	5	4	5	3	10	8	5	6	8	7
	standard error			35.6	30.4	20.4	13.5	2.3	31.2	20.2	35.3	10.2	27.1	22.1
		Method		MPA	AAo	SVC	DA	PBF^a^	DAo	UV	FO^b^	IVC_d_	IVC_p_^c^	DV
Mean Flow (% of CVO)	Human	2D PC MRI		56	41	29	40	15	54	29	29	–	–	–
	Mouse	ultrasound		52	48	23	36	21	42	14	31	–	30	10
	Sheep	microspheres		67	30	24	58	8	68	40	26	28	68	22
	Sheep	2D PC MRI		51	46	23	39	17	68	41	32	–	–	20
	Sheep	4D flow MRI		52	45	33	48	7	61	36	32	21	65	26

*Literature-based numbers for fetal blood distribution in human (from CMR* [9]*), mouse (from Doppler ultrasound* [7]*), sheep (from radiolabeled microspheres* [20]*), and sheep (from 2D PC CMR* [10]*)* versus *4D flow CMR data in sheep. Right hepatic vein flows, not presented here, were measured in 5 subjects*

*PBF* ^*a*^ *(mouse) calculated as MPA - DA. FO* ^*b*^ *(human, mouse, sheep – 2D PC CMR) calculated as AAo – PBF. IVC*_*p*_^*c*^ *(sheep – microspheres) calculated as UV + IVC*_*d*_

UV: umbilical vein; DV: ductus venosus; IVC_d_: distal inferior vena cava; IVC_p_: proximal inferior vena cava; SVC: superior vena cava; FO: foramen ovale; AAo: ascending aorta; DA: ductus arteriosus; DAo: descending aorta; MPA: main pulmonary artery; PBF: combined pulmonary blood flow

**Fig. 2:**
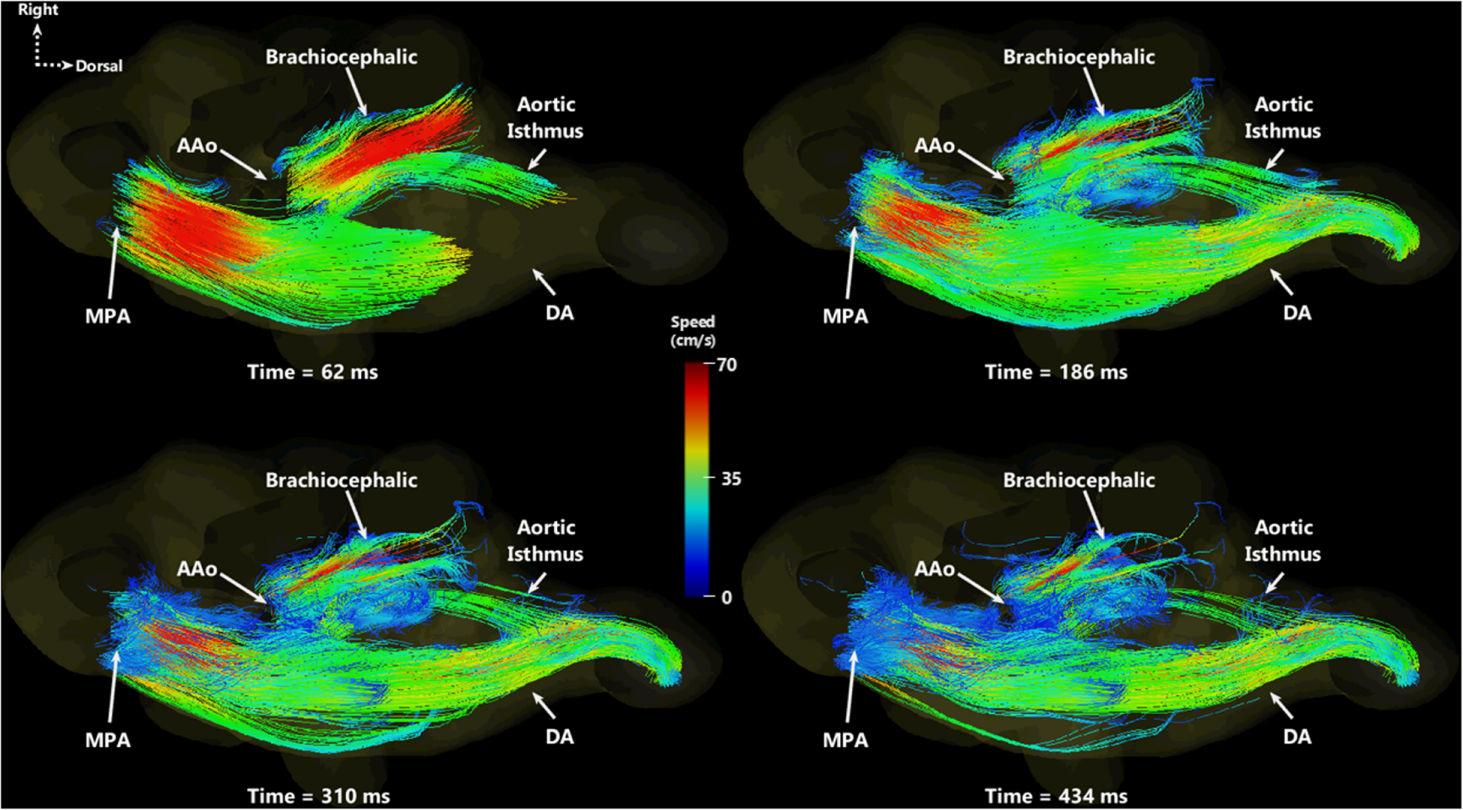
Individual time points from animated particle traces in a cranial view of cardiac structures. Particles are emitted from the main pulmonary artery (MPA) and ascending aorta (AAo), and color-coded based on speed of streaming blood. The majority of MPA flow bypasses the lungs (pulmonary arteries not visualized), shunting through the ductus arteriosus (DA) into the descending aorta and the rest of the body. The highest blood speed from the AAo passes into the brachiocephalic trunk. Animated versions of particle traces can be viewed over two cardiac cycles in Additional file 2: Movie 1

Particle traces depicting flows in the MPA and AAo are presented in Fig. 2. Qualitatively, the majority of traces leaving the MPA pass through the ductus arteriosus, bypassing the pulmonary circulation, while AAo blood branches into both the brachiocephalic trunk toward the brain and the DAo. An animated version of these particle traces can be viewed over two cardiac cycles in Additional file 2: Movie 1.

**Fig. 3:**
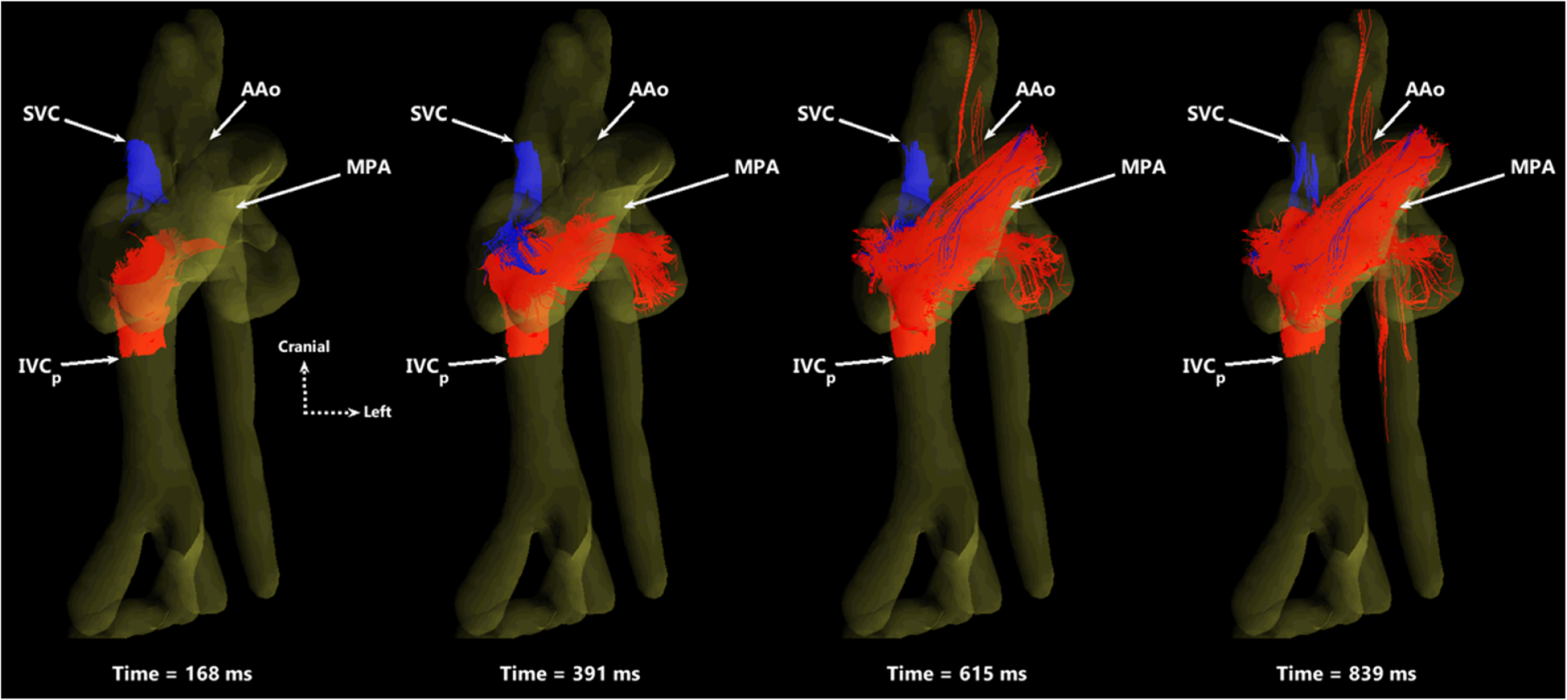
Particle traces in an oblique ventral view of SVC (blue) and IVC_p_ (red) return to the right side of the heart, shown at four time points over two cardiac cycles. The MPA receives blood from both vessels, serving as a conduit to the lungs and lower body. The AAo only receives IVC_p_ blood via right-to-left atrial shunting. Time-resolved particle trace animations over two cardiac cycles for this view can be found in Additional file 3: Movie 2. SVC: superior vena cava; IVC_p_: proximal inferior vena cava; AAo: ascending aorta; MPA: main pulmonary artery

Right heart inputs from the SVC (blue) and IVC_p_ (red) are shown in Fig. 3 as particle traces spanning two cardiac cycles. The MPA receives blood from both structures, yet the left side of the heart and AAo receive blood only from the lower body via the IVC_p_ and right-to-left shunting through the foramen ovale. An animated version of these particle traces can be viewed over two cardiac cycles in Additional file 3: Movie 2.

**Fig. 4:**
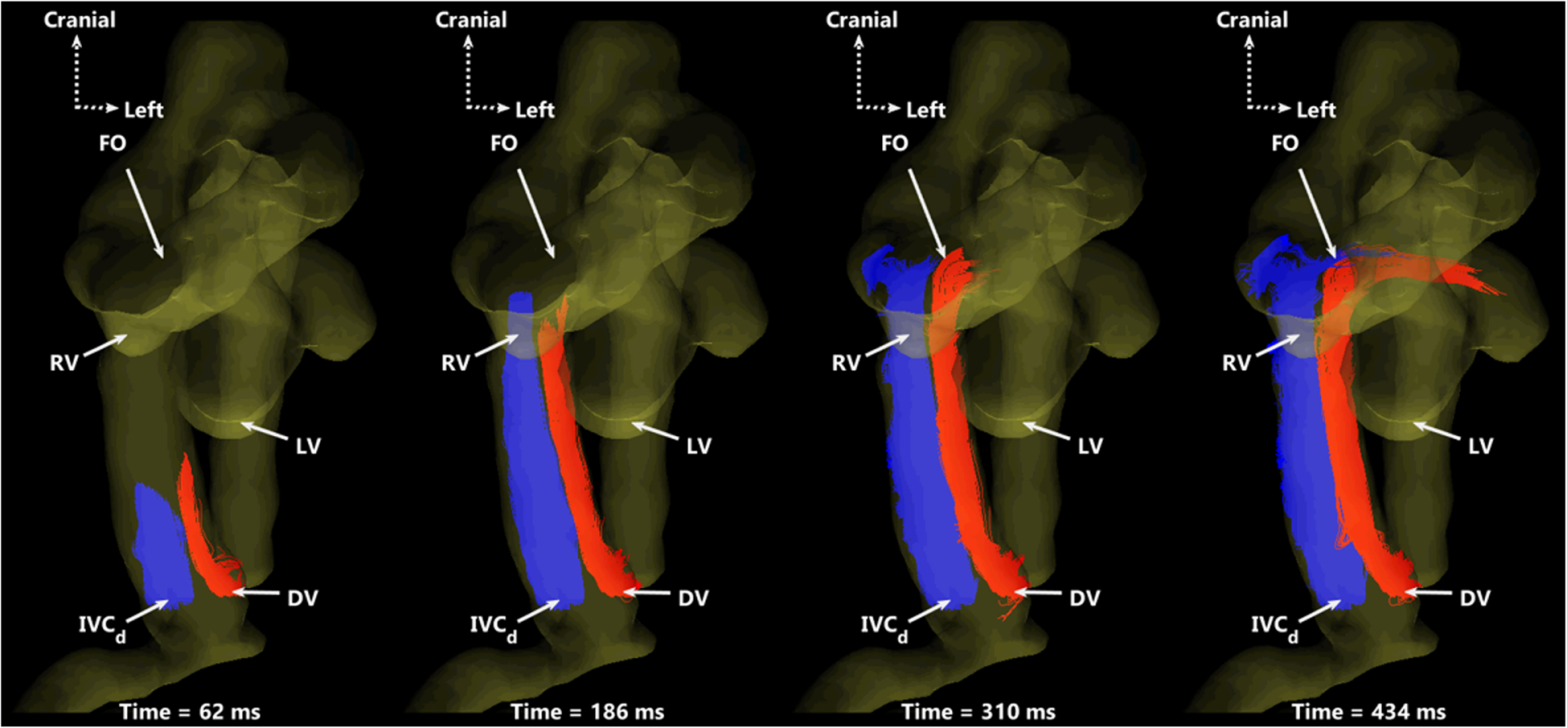
Particle traces in a ventral view showing preferential delivery of DV blood to the left side of the heart. DV (red) and IVC_d_ (blue) particles are shown at four time-points over one cardiac cycle. The two streams remain well-separated, with blood from the DV primarily entering the left heart while IVC_d_ blood passes into the right ventricle and main pulmonary artery. Particle trace movies over two cardiac cycles can be found in Additional file 4: Movie 3. DV: ductus venosus; IVC_d_: distal inferior vena cava; FO: foramen ovale; RV: right ventricle; LV: left ventricle

Particle traces of flow in the IVC_p_ are shown in Fig. 4, by simultaneously emitting particles from the ductus venosus (red) and the IVC_d_ (blue). Although these streams meet at the ductus venosus junction with the IVC_p_, they do not visibly mix as they approach the right atrium. Furthermore, note the preferential streaming of blood from the ductus venosus through the foramen ovale, to the left side of the heart. Animated particle traces over two cardiac cycles can be found in Additional file 4: Movie 3.

### Flow quantification

**Fig. 5:**
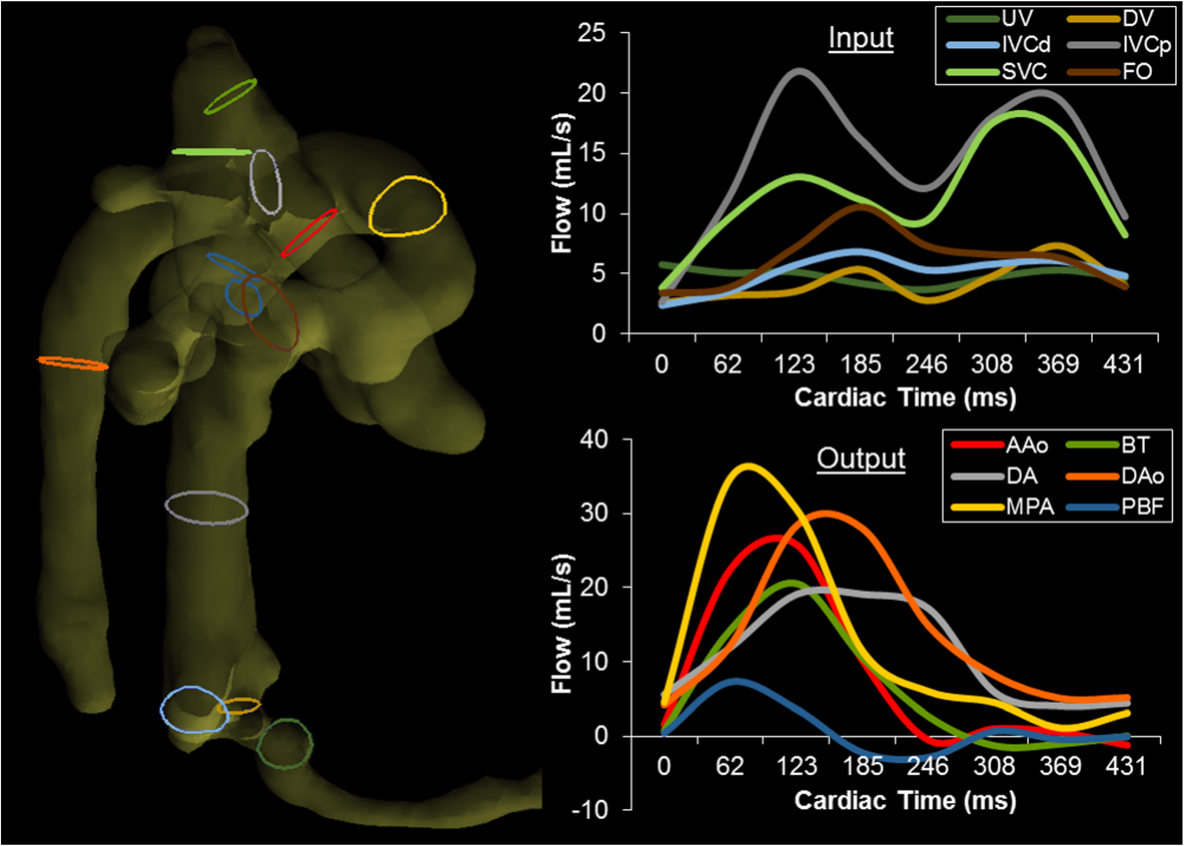
Left: Right lateral view of the segmented fetal vasculature for quantitation of blood flow volumes. Color-coded contours indicate locations of corresponding flow measurements at right. Right: Blood flow waveforms (flow in mL/s vs cardiac time) at all measurement locations, separated by input (top) and output (bottom). Cardiac inputs from the DV, IVC_d_, IVC_p_, and SVC display typical biphasic nature with greater pulsatility nearer to the heart, whereas UV flow appears nearly constant. Note UV flow was measured downstream from the junction of the typical 2 UVs found in fetal sheep. Outputs from the MPA, DA, AAo, BT, and DAo display large systolic peaks and low diastolic flows. BT and PBF display retrograde diastolic flow, likely indicative of runoff to the placenta through the aortic isthmus and DA. UV: umbilical vein; DV: ductus venosus; IVC_d_: distal inferior vena cava; IVC_p_: proximal inferior vena cava; SVC: superior vena cava; FO: foramen ovale; AAo: ascending aorta; BT: brachiocephalic trunk; DA: ductus arteriosus; DAo: descending aorta; MPA: main pulmonary artery; PBF: combined pulmonary blood flow

Figure 5 illustrates the locations of measurement contours within the fetal vasculature and their resulting blood flow waveforms, presented as cardiac inputs (top) and outputs (bottom) for clarity. These waveforms follow typical arterial and venous shapes, with venous waveforms showing increasing biphasic pulsatility closer to the heart (SVC, IVC_p_). Conversely, flow from the placenta through the umbilical vein is less affected by intracardiac pressures, appearing as a nearly constant flow.

4D flow CMR internal consistency across various cardiac structures showed percent differences of 14.2 ± 12.1% (*n* = 23 total locations), with average percent differences at specific locations of 20.3% (IVC_p_ = RHV + DV + IVC_d_; *n* = 7), 18.8% (SVC + IVC_p_ = MPA + FO; *n* = 4), 7.4% (MPA = DA + PBF; n = 4), and 10.1% (IVC_p_ = DAo; *n* = 8).

**Fig. 6:**
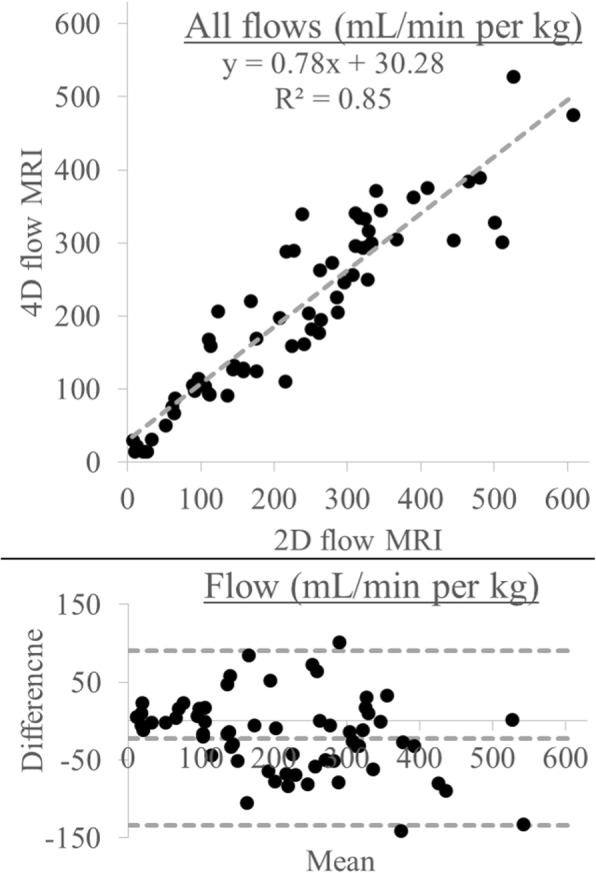
Linear regression (left) and Bland-Altman (right) analysis comparing 2D PC CMR and 4D flow MRI, with all vessels and subjects combined. 4D flow CMR shows good correlation with 2D PC CMR (R^2^ = 0.85, slope = 0.78 with 95% confidence interval = [0.69, 0.86])). The calculated Bland-Altman bias = − 21.88 (limits of agreements = [− 133.82, 90.05] mL/min per kg) was considered significant (*p* = 0.009)

2D PC CMR versus 4D flow CMR yielded 65 direct flow comparisons across all subjects. Results for linear regression and Bland-Altman analysis are shown in Fig. 6, demonstrating strong correlation (R^2^ = 0.85) but with systematic underestimation using 4D flow (slope = 0.78, 95% confidence interval = [0.69, 0.86]). This resulted in a statistically significant (*p* = 0.009) bias of − 21.9 mL/min per kg (limits of agreement = [− 133.8, 90.1]). These results are further parsed and color-coded by individual vessels in Additional file 5: Fig. S2, and all vessels are individually visualized using boxplots in Additional file 6: Fig. S3..

Table 3 provides mean indexed flows in mL/min per kg fetal mass, and these same values as %CVO. Human [9], mouse [7], and sheep [10, 20] reference data are also included for comparison with values from this 4D flow CMR sheep fetus experiment.

## Discussion

Here we use 4D flow CMR for evaluation of the fetal circulation in a sheep model of human pregnancy, and present compelling evidence of parallel streams of blood passing up the IVC and through the fetal heart. This streaming is a well-known mechanism for oxygen delivery to the developing heart and brain, and the findings here are supported by previous studies [1, 6]. To determine the path of oxygenated versus deoxygenated blood, Edelstone and Rudolph [1] injected radiolabeled microspheres into the major vessels feeding the heart with oxygenated (ductus venosus) and deoxygenated (IVC_d_) blood and counted the distribution of their downstream tissue deposition in the sheep fetus. This approach has also been used to determine distribution of fetal cardiac output in response to acute [22] and chronic [15] hypoxemia and hormonal regulation [23–25]. The fetal circulation has also been probed with more conventional diagnostic imaging, including multimodal ultrasound to examine 2D projections of ductus venosus and foramen ovale shunting [6], Doppler ultrasound to measure ductus venosus waveforms in acute hypoxemia [26], and 2D PC CMR to measure distribution of cardiac output in anesthetized sheep fetuses [10].

Complete cardiovascular segmentation was achieved in 5/10 cases. Cases that could not be fully segmented were a result of either incomplete 4D flow volume coverage of the fetal vasculature or artifact from maternal respiration. In two cases the prescribed image volume did not cover the umbilical vein to the aortic arch, while in three cases segmentation of smaller vessels failed due to residual image artifact attributed to maternal respiratory motion. In these cases, only large, straight vessels were identified and measured.

Using volumetric velocity-mapping CMR and corresponding particle trace evolution, the results presented here are the first to visualize ductus venosus streaming in a 3D time-resolved manner. By direct observation of these complex distributions using 4D flow CMR, a more complete representation and understanding of normal fetal growth may be achieved, and how pathologies that influence this flow, such as congenital heart disease, alter development.

In addition to the qualitative assessment of the fetal cardiac circulation, 4D flow CMR can be used as a tool for direct measurement of flow as it traverses all major cardiac vessels and shunts. Due to certain vessels remaining unidentified, conservation of mass could not be performed across all junctions in all fetal subjects. For those that were measured, the percent differences across cardiac structures fall into a normal range for 4D flow CMR previously reported at this level of spatial and temporal resolution. Wentland et al. used 4D flow CMR in the adult abdominal aorta and renal arteries (which included 1.32 mm isotropic resolution, ~ 71.5 ms temporal resolution, and respiratory gating), reporting internal consistency of 12.6 ± 8.8%. [27]. Additionally, using 4D flow CMR in the pregnant rhesus macaque, Macdonald et al. have shown internal consistency on the order presented here, specifically: 15% in the uterine arteries, 8% between DAo and IVC_p_ measurements, and 15% in the umbilical cord [28]. Given the smaller intracardiac fetal vessels measured in this study and lack of respiratory gating, one would expect a worse consistency. Nevertheless, these results lend confidence to the internal consistency of measurements presented here.

Direct statistical comparison of 4D flow CMR to 2D PC CMR in fetal vessels was inspired by previous work in human in vivo fetal measurements [8, 9]. Moreover, the use of 2D PC CMR as a standard for these measurements is supported by its ubiquitous clinical use and repeatability in vessels whose diameters are on the order of the adult internal carotid artery [29]. Despite establishing good correlation between 4D flow CMR and 2D PC CMR, the 4D flow values were significantly lower. This underestimation of the 4D flow values may result from four main factors. First, there are user-dependent factors inherent to both techniques, including manual drawing of regions of interest, which may introduce measurement variability. Second, lower in-plane spatial resolution in the 4D flow CMR scans (1.2–1.5 × 1.2–1.5 mm^2^ versus 1.0 × 1.0 mm^2^) likely resulted in partial volume effects at the vessel edge. It has previously been reported that accurate blood volume flow rates can be determined with ~ 3 pixels per vessel diameter [30]. As a check of this requirement, the minimum vessel area across all 4D flow measurements (RPA = 21.5 mm^2^) was assumed to have a circular contour. This corresponds to an approximate diameter of 3.5 voxels at 1.5 × 1.5 × 1.5 mm^3^ resolution. Third, flow waveform data points are less frequently sampled due to a roughly 2 times lower temporal resolution from 4D flow CMR (45–62 ms versus 24–33 ms). These temporal resolution differences likely contribute to the bias seen here, though may be dependent on the shape of the flow waveform [31]. As shown in the vessel color-coded Bland-Altman analysis and boxplots (Additional file 5: Figure. S2 and Additional file 6: Figure. S3), this is particularly evident in larger vessels such as the MPA, ductus arteriosus, and DAo. Finally, as 4D flow CMR was performed at the end of lengthy scans, maternal cardiac output may have been lowered due to the cumulative effect of hours of anesthesia [32].

Indexed flows (mL/min per kg) determined with 4D CMR were on the order of those previously reported in humans [8, 9] and sheep [10, 20], and far lower than those presented in mice [7]. Variability within and between these groups indicates the need for further studies and more advanced tools, such as 4D flow CMR, to probe a regional evaluation of fetal circulation that can be applied across species. Some variation may arise from the need to anesthetize animals for CMR studies, however fetal blood gases were in the normal range in the current study. Despite interspecies variation and potential blood flow perturbations from anesthesia, it is important to point out the similarity in %CVO across species for each measured vessel. This, coupled with the moderate internal consistency discussed above, offers assurance for the capability of 4D flow CMR to characterize the regional distribution of flow across fetal structures of varying size.

This study had certain limitations. First, maternal respiration caused considerable variability in image quality and inconsistent identification and segmentation of vessels of interest. Respiratory navigator gating was turned off to reduce total acquisition time, at the cost of motion artifact and noise. Single-slice scans, such as 2D PC CMR, do not suffer as much from respiratory motion as they can be tailored to be frequency encoded along the direction of respiratory motion. Future work will explore the use of 4D flow CMR acquisitions and reconstructions that measure and compensate for this respiratory motion and residual bulk fetal motion. Another limitation lies in the small cohort with full anatomical view and insufficient statistical power to evaluate clinically relevant covariates. Because this study had low numbers, no inter-group analyses (e.g. between fetal size or oxygen status) could be performed and all fetal sheep were grouped into one cohort. Future experiments will allow for these factors to be systematically explored.

## Conclusion

In conclusion, normal fetal development relies on a complex circulatory system that is notably different than post-natal circulation. Improving the understanding of fetal blood flow patterns has inherent medical and educational relevance, especially in studying normal physiological circulation. The work presented here provides insight into the physical mechanism by which the fetus preferentially supplies oxygen-rich blood to essential organs such as the brain and the heart, and lays a foundation for preclinical studies of fetal cardiovascular physiology using volumetric PC CMR.

## Additional files

Additional file 1: **Figure S1.** Normal course of fetal circulation with locations of conservation of mass calculation overlaid as colored segments: IVC_p_ = RHV + DV + IVC_d_ (yellow, *n* = 7); SVC + IVC_p_ = MPA + FO (green, *n* = 4); MPA = DA + PBF (red, n = 4); IVC_p_ = DAo (blue, *n* = 8). DV: ductus venosus; IVC_d_: distal inferior vena cava; IVC_p_: proximal inferior vena cava; SVC: superior vena cava; FO: foramen ovale; MPA: main pulmonary artery; PBF: pulmonary blood flow; DA: ductus arteriosus; DAo: descending aorta. (TIF 7890 kb)

Additional file 2: **Movie 1.** Animated particle traces in a cranial view of cardiac structures in a sheep fetus, shown over two cardiac cycles. Particles are emitted from the main pulmonary artery (MPA) and ascending aorta (AAo), and color-coded based on speed of streaming blood. (GIF 13701 kb)

Additional file 3: **Movie 2.** Animated particle traces in an oblique ventral view, shown over two cardiac cycles. Particles are emitted from the superior vena cava (SVC, blue) and proximal inferior vena cava (IVC_p_, red). MPA: main pulmonary artery; AAo: ascending aorta. (GIF 5828 kb)

Additional file 4: **Movie 3.** Animated particle traces in ventral view of cardiac structures in a sheep fetus, shown over two cardiac cycles. Traces are emitted from the ductus venosus (DV, red) and from the distal inferior vena cava (IVC_d_, blue). RV: right ventricle; LV: left ventricle; FO: foramen ovale. (GIF 6613 kb)

Additional file 5: **Figure S2.** Linear regression (left) and Bland-Altman (right) analysis comparing 2D PC MRI and 4D flow MRI, with individual color-coded vessels across all subjects. (TIF 224 kb)

Additional file 6: **Figure S3.** Boxplot comparison between 2D PC MRI (blue boxes) and 4D flow MRI (black boxes) for each individual vessel. Greater underestimation of 4D flow MRI is seen in the DAo and DA. (TIF 1826 kb)

## Abbreviations

3D: three dimensional
4D: four dimensional
AAo: ascending aorta
BT: brachiocephalic trunk
CMR: cardiovascular magnetic resonance
CVO: combined ventricular output
DA: ductus arteriosus
DAo: descending aorta
DV: ductus venosus
FO: foramen ovale
IVC_d_: distal inferior vena cava
IVC_p_: proximal inferior vena cava
LA: left atrium/left atrial
LPA: left pulmonary artery
LV: left ventricle/left ventricular
MPA: main pulmonary artery
PBF: pulmonary blood flow
PC: phase contrast
RA: right atrium/right atrial
RHV: right hepatic vein
RPA: right pulmonary artery
RV: right ventricle/right ventricular
SVC: superior vena cava
TE: echo time
TR: repetition time
UV: umbilical vein
VENC: velocity encoded
