# Supplementary figures and images for: Fetal hemodynamics and cardiac streaming assessed by 4D flow cardiovascular magnetic resonance in fetal sheep
Source: J Cardiovasc Magn Reson. 2019 Jan 21;21:8. doi: 10.1186/s12968-018-0512-5 (PMC6340188; doi:10.1186/s12968-018-0512-5)

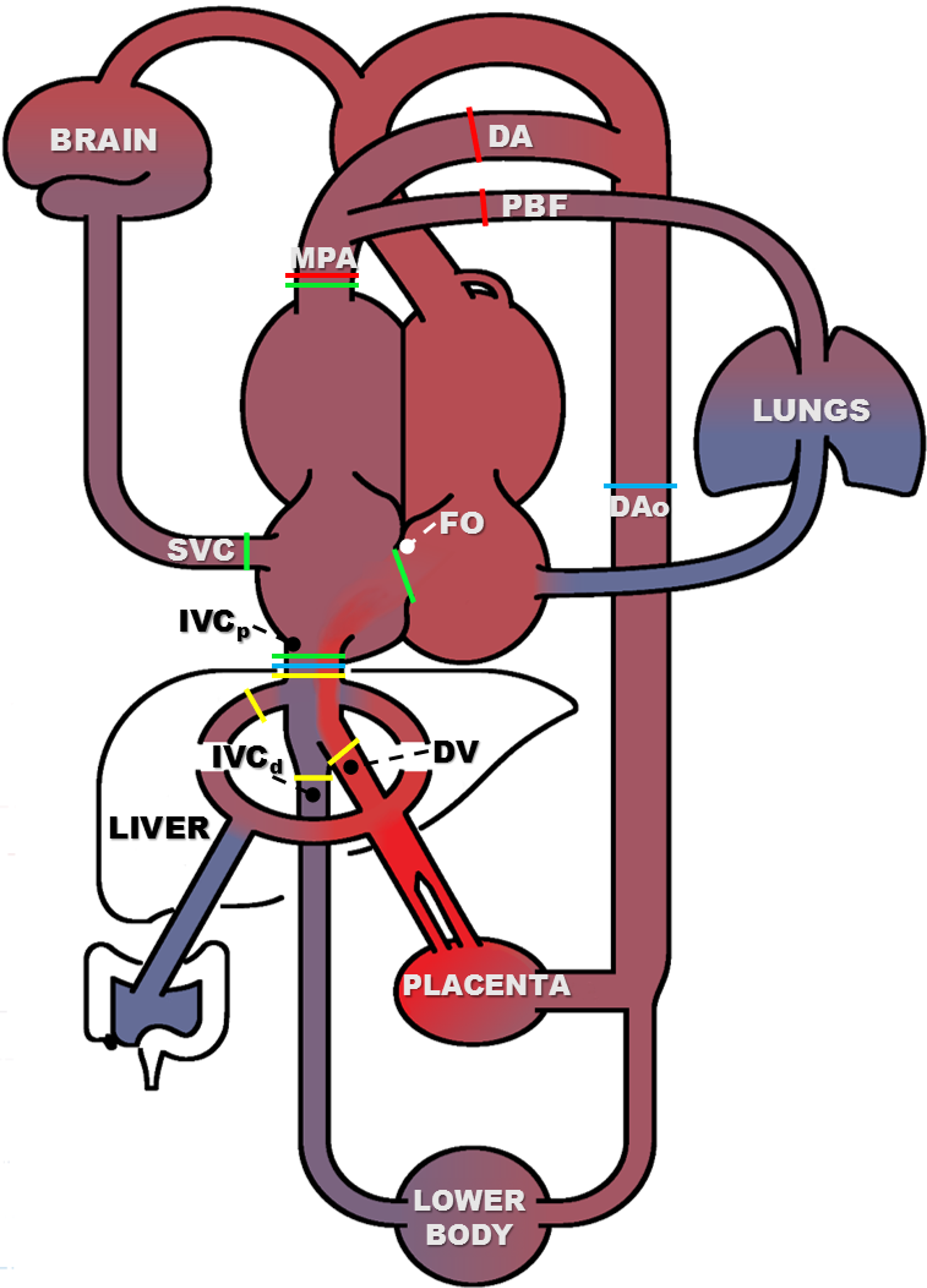

Supplement: Supplementary file 1 — Figure S1. Normal course of fetal circulation with locations of conservation of mass calculation overlaid as colored segments: IVCp = RHV + DV + IVCd (yellow, n = 7); SVC + IVCp = MPA + FO (green, n = 4); MPA = DA + PBF (red, n = 4); IVCp = DAo (blue, n = 8). DV: ductus venosus; IVCd: distal inferior vena cava; IVCp: proximal inferior vena cava; SVC: superior vena cava; FO: foramen ovale; MPA: main pulmonary artery; PBF: pulmonary blood flow; DA: ductus arteriosus; DAo: descending aorta. (TIF 7890 kb) [file 12968_2018_512_MOESM1_ESM.tif]

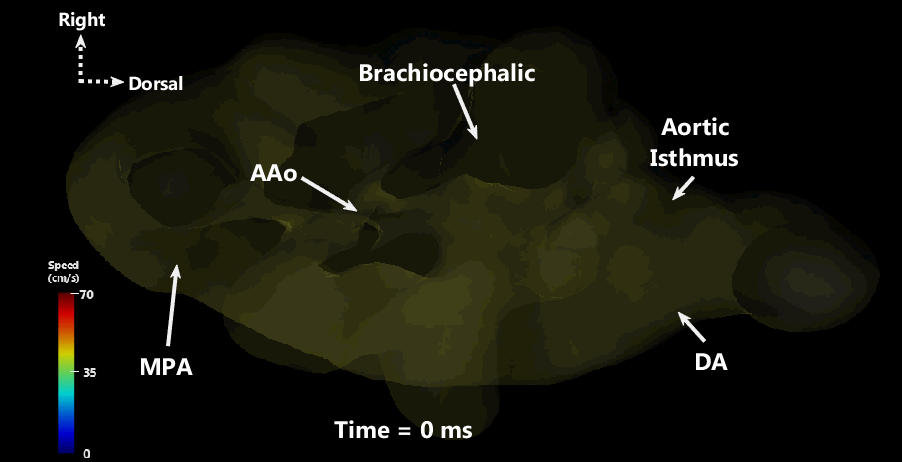

Supplement: Supplementary file 2 — Movie 1. Animated particle traces in a cranial view of cardiac structures in a sheep fetus, shown over two cardiac cycles. Particles are emitted from the main pulmonary artery (MPA) and ascending aorta (AAo), and color-coded based on speed of streaming blood. (GIF 13701 kb) [file 12968_2018_512_MOESM2_ESM.gif]

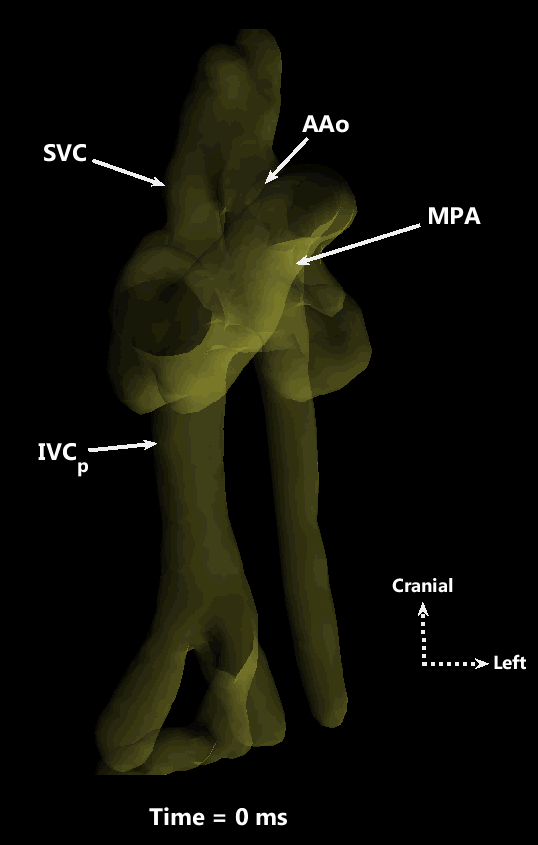

Supplement: Supplementary file 3 — Movie 2. Animated particle traces in an oblique ventral view, shown over two cardiac cycles. Particles are emitted from the superior vena cava (SVC, blue) and proximal inferior vena cava (IVCp, red). MPA: main pulmonary artery; AAo: ascending aorta. (GIF 5828 kb) [file 12968_2018_512_MOESM3_ESM.gif]

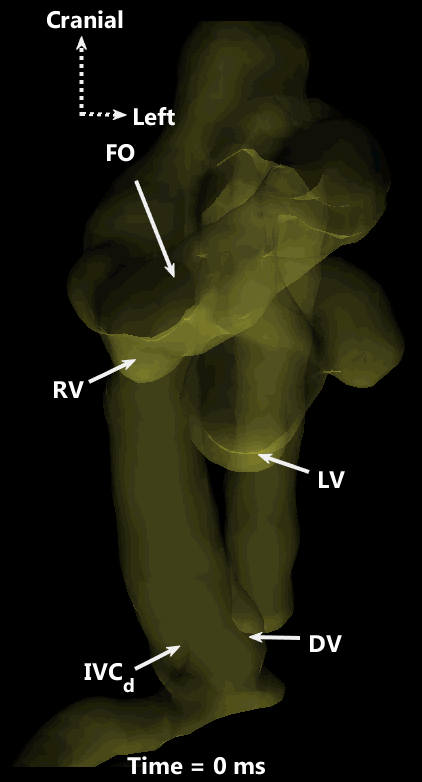

Supplement: Supplementary file 4 — Movie 3. Animated particle traces in ventral view of cardiac structures in a sheep fetus, shown over two cardiac cycles. Traces are emitted from the ductus venosus (DV, red) and from the distal inferior vena cava (IVCd, blue). RV: right ventricle; LV: left ventricle; FO: foramen ovale. (GIF 6613 kb) [file 12968_2018_512_MOESM4_ESM.gif]

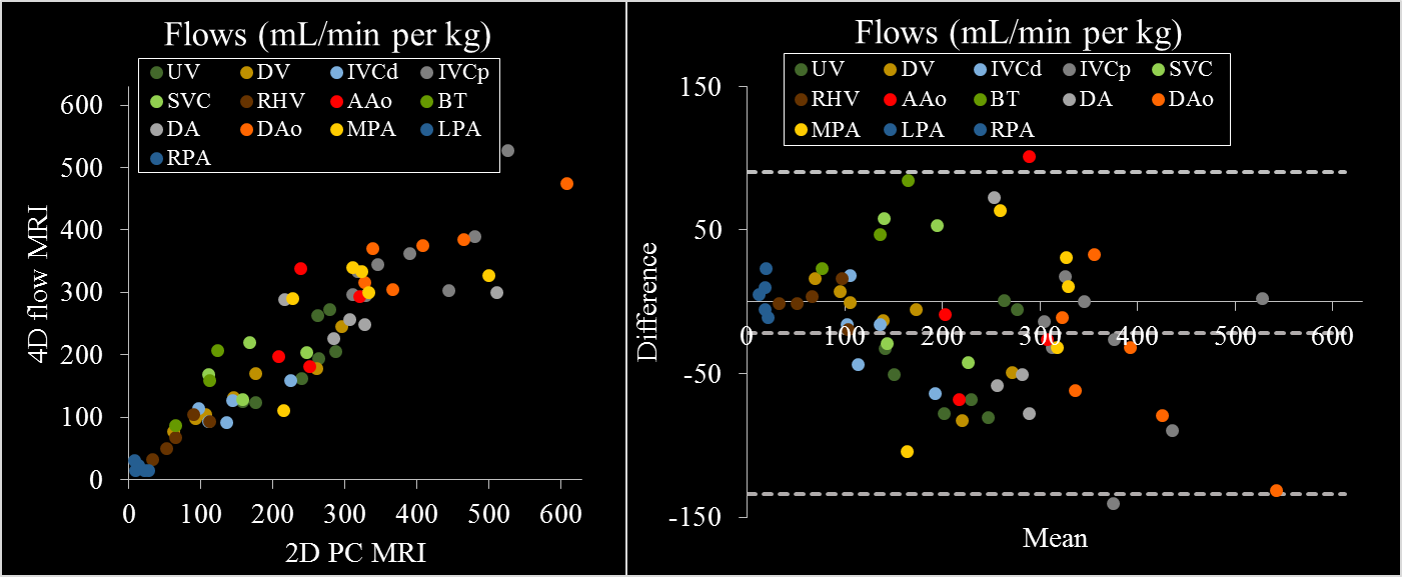

Supplement: Supplementary file 5 — Figure S2. Linear regression (left) and Bland-Altman (right) analysis comparing 2D PC MRI and 4D flow MRI, with individual color-coded vessels across all subjects. (TIF 224 kb) [file 12968_2018_512_MOESM5_ESM.tif]

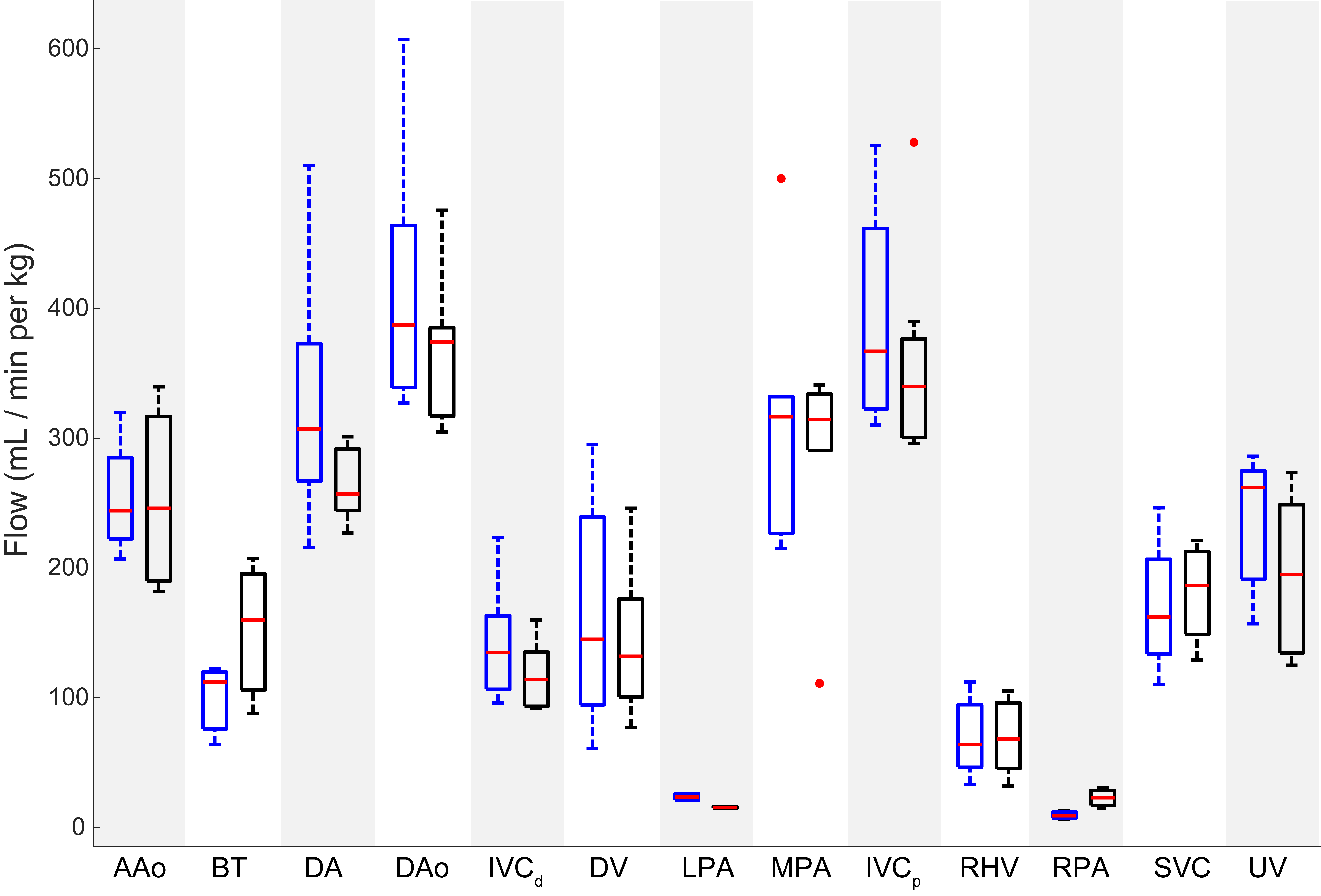

Supplement: Supplementary file 6 — Figure S3. Boxplot comparison between 2D PC MRI (blue boxes) and 4D flow MRI (black boxes) for each individual vessel. Greater underestimation of 4D flow MRI is seen in the DAo and DA. (TIF 1826 kb) [file 12968_2018_512_MOESM6_ESM.tif]
